# Three slice myocardial coverage using non-ECG-triggered perfusion imaging with integrated T1 mapping for quantifying myocardial blood flow

**DOI:** 10.1186/1532-429X-17-S1-Q61

**Published:** 2015-02-03

**Authors:** David Chen, Behzad Sharif, Janet Wei, Xiaoming Bi, Reza Arsanjani, Louise E Thomson, Noel C Bairey Merz, Daniel S Berman, Debiao Li

**Affiliations:** 1 BIRI, Cedars Sinai Hospital, Los Angeles, CA, USA; 2 Biomedical Engineering, Northwestern University, Evanston, IL, USA; 3 S. Mark Taper Foundation Imaging Center, Cedars Sinai Medical Center, Los Angeles, CA, USA; 4 MR R&D, Siemens Healthcare, Los Angeles, CA, USA; 5 Barbra Streisand Women's Heart Center, Cedars Sinai Medical Center, Los Angeles, CA, USA; 6 Bioengineering, University of California, Los Angeles, Los Angeles, CA, USA

## Background

A single-bolus non-ECG-triggered method was previously proposed to reduce the complexity of quantitative myocardial perfusion imaging [1]. Compared to conventional dual-bolus method, the arterial input function (AIF) is derived from imaging data, eliminating the need for an additional scan. The imaging workflow is further simplified by retrospectively determining the proper reconstruction window, eliminating the dependency on ECG triggering. However, only a single slice was acquired. In this work, we expand upon the previous technique to 3 slices using highly constrained back projection (HYPR) reconstruction to achieve clinically acceptable myocardial coverage.

## Methods

Data was acquired continuously with no ECG-triggering. 26 projections per slice were acquired following each SR-preparation in an interleaved manner. HYPR was used to reconstruct images with 5-fold undersampling. A sliding window was used to produce series of low resolution images in the basal slice with a temporal resolution of 44 ms. Triggering signal was derived retrospectively from the mean signal intensity in an ROI drawn around the heart. AIF was estimated using single-cardiac cycle T1 mapping [1]. In short low resolution images reconstructed from subsets of projections are used to sample the T1 relaxation. The image signal intensities of the ventricular blood pool are then fit to the Bloch equation to solve for T1. T1 values are then converted to contrast agent concentrations.

Ten healthy volunteers underwent rest perfusion MRI on a Siemens 3T Verio. A dual-bolus protocol was performed using a conventional clinical Cartesian sequence for comparison [3]. Ten minutes following the dual-bolus scan, a second first-pass scan was performed using the proposed method. The mean myocardial blood flow (MBF) in each subject was compared using a two-sided Student's t-test at a p=0.05 significance level. In a 2^nd^ study, another 12 healthy volunteers underwent stress-rest studies using the proposed method. Mean myocardial perfusion reserve (MPR) was compared with previously published values.

## Results

Mean MBF values found using the ECG-triggered dual-bolus method and the non-ECG-triggered integrated T1 mapping method were 0.82 ± 0.21 and 0.76 ± 0.13 ml/min/g, respectively. There was no significant difference (p = 0.45) between them. The mean rest and stress MBF and MPR was 0.86±0.5, 3.91±1.1 ml/min/g, and 4.31±1.3, respectively.

## Conclusions

A 3-slice, non-ECG-triggered, single-bolus quantitative perfusion MR method with integrated T1 mapping for AIF measurement produces similar MBF as the reference dual-bolus method with comparable ventricular coverage. Mean MPR is similar to those reported in literature. The proposed non-ECG-triggered technique improves ease-of-use, and has the potential to improve robustness to arrhythmias. This method may improve the clinical feasibility of quantitative myocardial perfusion imaging.

## Funding

NIH grant numbers T32 EB51705 and RO1 EB002623, NIBIB grant number EB002623, AHA Scientist Development Grant 14SDG20480123, GCRC grant MO1-RR00425, and Edythe L. Broad Women's Heart Research Fellowship UN55ES6580F.

**Figure 1:**
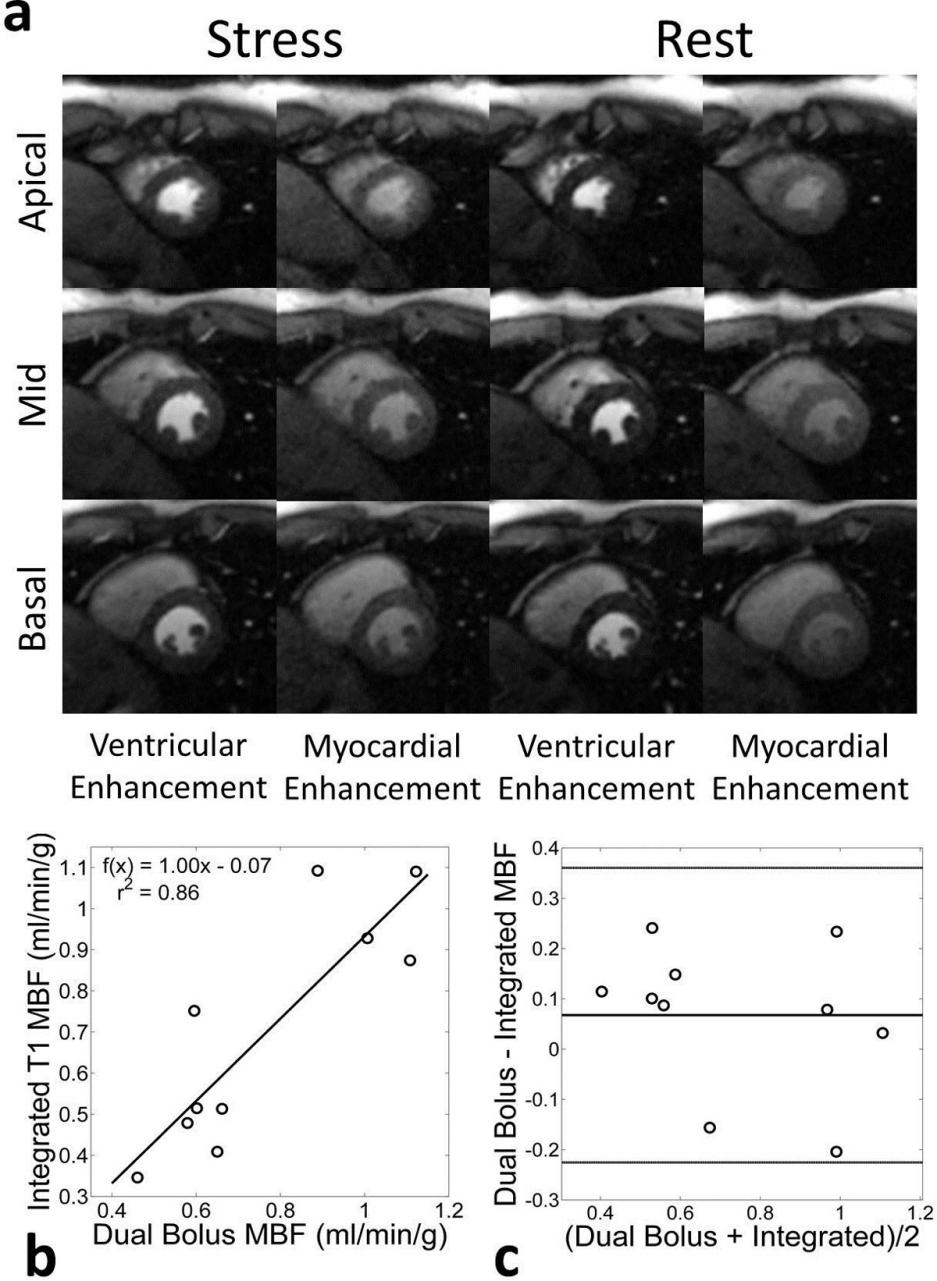
Comparison of proposed single-bolus non-ECG-triggered and conventional dual-bolus ECG-triggered methods. (a) Comparison of example ECG-triggered Cartesian and the non-ECG-triggered radial images in a rest perfusion study. Because ECG-triggered Cartesian images are acquired sequentially, they fall during different cardiac phases (mid-systole in the apical compared to early-diastole of the basal slice). (b) Correlation of mean MBF found between the two techniques (r^2^ = 0.86). (b) A Bland-Altman plot comparing the two methods.

**Figure 2:**
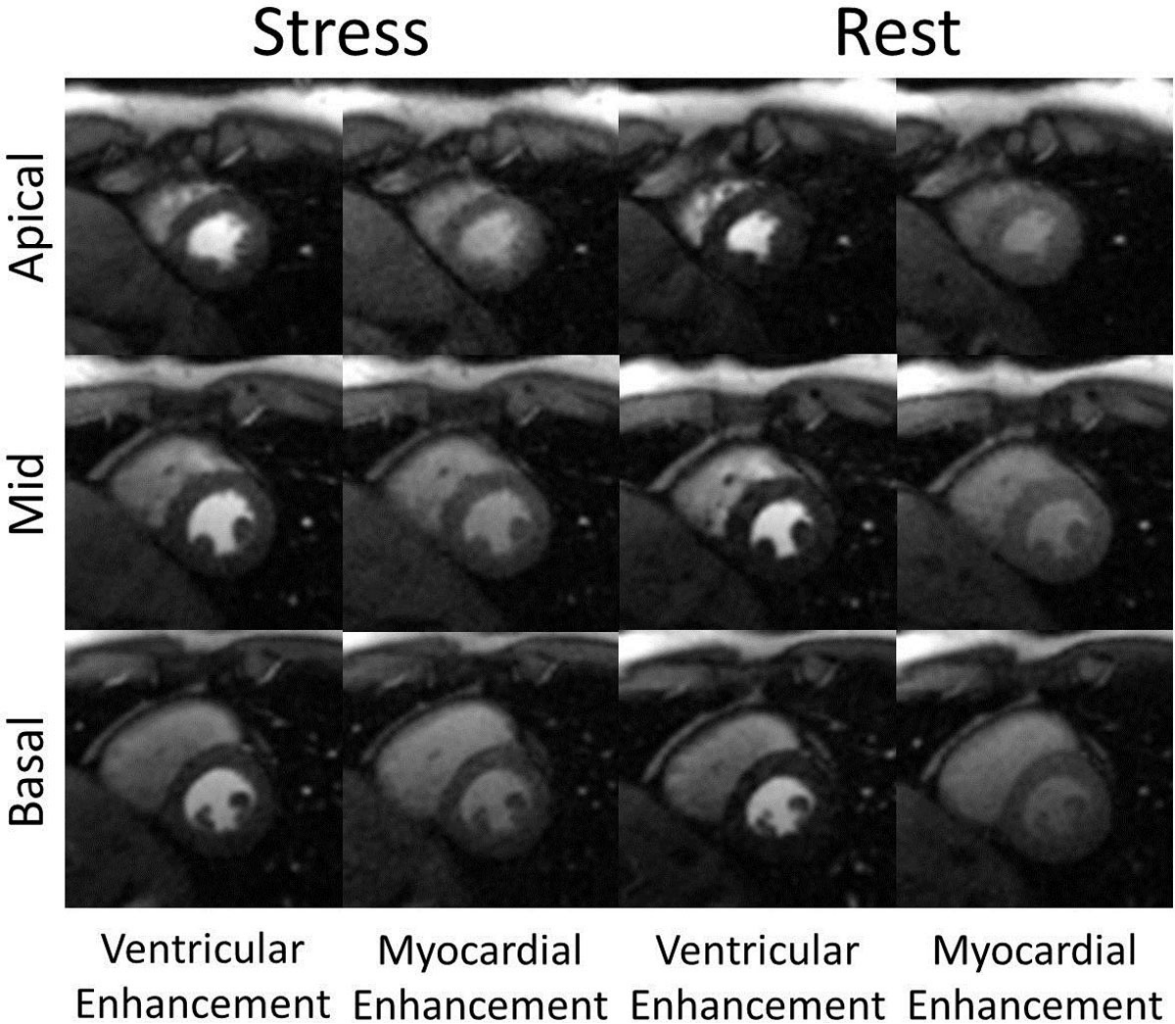
Example non-ECG-triggered stress-rest perfusion images from a healthy volunteer study. Each image was reconstructed from 26 projections. Stress images were acquired first. All images were acquired during systole to maximize myocardial thickness. Images of peak ventricular enhancement and peak myocardial enhancement are shown.
